# Origin and Nature of Falling Stars

**Published:** 1834

**Authors:** 


15. *Origin and Nature of the Meteors of the* 13*th November*, 1833.—In our 27th number, we gave a detailed historical account of these meteors, not venturing on a single speculation concerning their origin or nature—matters that belong to the science of physical astronomy, which we do not cultivate. Among the most interesting of the accounts which we then embodied, was that of the learned professor Olmsted of Yale College. In the April number, for the present year, of Silliman's American Journal, a work which does honor to our country, we observe an elaborate paper by the same ingenious professor, on the source and constitution of those shooting stars. Without attempting its analysis, we shall present our readers with the conclusions to which that gentleman has come; omitting the facts and reasoning by which they are supported:

1. "The meteors of November 13, (1833) had their origin beyond the limits of our atmosphere."

Of this we think there can be no doubt. The reader must not confound the place of *origin*, with the place of *luminous exhibition*.

2. "The height of the place whence the meteors emanated, above the surface of the earth was about 2238 miles."

Notwithstanding the apparent precision of this statement, which presents the mean term of several estimates, it must be considered as nothing more than an approximation to the truth.

3. "The Meteors fell towards the earth, being attracted to it by the force of gravity."

4. "The Meteors fell towards the earth in straight lines, and in parallel directions, which within considerable distances, were nearly parallel with each other."

It must be recollected that parallel lines seen in very distant perspective, appear at last to unite in a focus, and such was the spot in the heavens from which the shooting stars seemed to radiate. It was near the constellation Leo.

5. "The Meteors entered the earth's atmosphere with a velocity of about four miles per second."

This would be the velocity they would acquire in falling, from a state of previous rest in reference to the earth, to the limits of the atmosphere, 50 miles from its surface, as determined by calculation.

6. "The Meteors consisted of combustible matter, and took fire and were consumed in traversing the atmosphere."

Hence while their origin was beyond the atmosphere we only saw them within it. Their combustion was matter of observation. They were probably ignited by the compression they exerted on the air, forcing out its caloric in sufficient quantities to ignite them. That they were consumed was matter of pure observation for many of them disappeared in luminous clouds which were wafted by the wind, and none of them were seen on the surface of the earth.

7. "Some of the Meteors must have been bodies of great size."

8. "The Meteors were combustible bodies and were constituted of light and transparent materials."

Their combustibility was demonstrated by their burning. If they had not been light they would have made their way like ærolites, through the atmosphere to the surface of the earth. If they had not been transparent they would have been visible, or rather the cloud or nucleus from which they were detached, would have been visible, at the height of 2 or 3000 miles, by reflecting the light of the sun.

Professor Olmsted goes on to consider the question whether the mass from which the meteors were detached, belonged to and revolved round the earth, and comes to the conclusion, from its occupying one spot in the heavens for several hours, while the earth was rotating, that it was not a satellite. He is also convinced, that it could not have been at rest and approached, on the 13th of November, by the earth, as such a body could not have remained at rest within the solar system; nor could it have been in motion, in a different direction from that which the earth was moving, as they would soon have separated—seeing, that in the eight hours during which the meteoric shower lasted, the earth moved in its orbit through the space of nearly 550,000 miles. Hence he comes to the conclusion, "that the body was pursuing its way along with the earth round the sun;" and from other considerations arrives at the further conclusion, "that it revolves round the sun in an illiptical orbit; but little inclined to the plane of the ecliptic, and having its aphelion near to the orbit of the earth." In regard to its periodic time round the sun, he thinks from the best data within his reach, that it is "nearly six months, and that its perihelion is a little below the orbit of Mercury." Thus he regards it, in fact, as a comet, and concentrates his opinions of its astronomic relations into the final proposition—

"That the Meteors of November 13th, consisted of portions of the extreme parts of a nebulous body, which revolves round the sun in an orbit interior to that of the earth, but inclined to the plane of the ecliptic, having its aphelion near to the earth's path, and having a periodic time of 182 days, nearly."

Believing as we constantly have, that these meteors were not of atmospheric origin, we take pleasure in presenting to our readers, but few of whom, we suppose, will meet with Professor Olmsted's paper, the results of his scientific and ingenious inquiries into this *luminous* but *obscure* phenomenon.

